# Species-specific cadmium and lead tolerance in four *Eucalyptus* seedlings: implications for phytoremediation of metal-contaminated soils

**DOI:** 10.3389/fpls.2026.1908520

**Published:** 2026-08-21

**Authors:** Gunjan Patil, Muthusamy Palani Divya, Abhishek Maitry, Preety Dubey, Damini Sharma, Ramesh Sahu, Wagmare Balraju

**Affiliations:** 1 Forest College and Research Institute, Tamil Nadu Agricultural University, Coimbatore, Tamil Nadu, India; 2 Department of Forestry, Wildlife and Environmental Sciences, Guru Ghasidas Vishwavidyalaya, Bilaspur, Chhattisgarh, India; 3 School of Studies in Forestry & Wildlife, Shaheed Mahendra Karma Vishwavidyalaya, Jagdalpur, Chhattisgarh, India; 4 Department of Forest Biology and Tree Improvement, Mahatma Gandhi University of Horticulture and Forestry, Durg, Chhattisgarh, India; 5 Birbal Sahni Institute of Palaeosciences, Lucknow, Uttar Pradesh, India

**Keywords:** cadmium, *Eucalyptus* species, heavy metal, lead, phytotoxicity, tolerance index

## Abstract

**Introduction:**

Phytoremediation using fast-growing tree species has emerged as a sustainable approach for restoring metal-contaminated soils. However, comparative information on species-specific responses to metal stress tolerance remains limited.

**Methods:**

This study evaluated chlorophyll content, growth performance, and biomass allocation, including metal tolerance efficiency, of four *Eucalyptus* species (i.e., *E. tereticornis, E. camaldulensis, E. globulus*, and *E. citriodora*) under varying concentrations of Cadmium (Cd, 25, 50, and 100 mg kg⁻¹) and Lead (Pb, 100, 125, and 250 mg kg⁻¹). A factorial, completely randomized pot experiment was conducted under controlled conditions, using sterilized red soil as the medium for 120 days. Plant growth performance and metal tolerance efficiency were measured and analyzed using ANOVA followed by *post hoc* comparison.

**Results:**

Cd exhibited significantly greater phytotoxicity than Pb across species and parameters studied. Marked species-specific differences in metal tolerance were observed in *Eucalyptus citriodora*, outperforming other species in maintaining superior growth performance and metal tolerance even at the highest metal concentrations (Cd 100 mg kg⁻¹). However, *E. camaldulensis* was the species most sensitive to Cd stress. Hierarchical clustering rank based on superior tolerance characteristics of species was: *E. citriodora > E. camaldulensis > E. tereticornis > E. globulus*.

**Discussion:**

It was concluded that *Eucalyptus citriodora* was the most promising candidate for future phytoremediation studies of Cd- and Pb-contaminated soils due to its superior metal tolerance ability as reflected by sustained growth performance and chlorophyll retention of the species. The species can be recommended for restoration programs and sustainable management of metal-polluted areas based on performance at the initial phase.

## 1 Introduction

Across the globe, anthropogenic activities have progressively loaded soils with toxic heavy metals, transforming productive lands into reservoirs of persistent pollutants that threaten food security, human health, and ecosystem stability (Shakti and Pandey, 2024). Recent meta-analyses confirm that Cd and Pb remain the most widespread and hazardous metal contaminants globally, with arbuscular mycorrhizal fungi emerging as a promising bioremediation tool for mine-affected soils (Banerjee et al., 2025). In natural terrestrial environments, soils contain intrinsic levels of trace elements that reflect the biogeochemical characteristics of their parent material (Alloway, 2013; Kabata-Pendias, 2000). Elements such as Fe, Zn, Co, Cu, and Mn serve as essential micronutrients, playing critical roles in enzymatic regulation, redox reactions, and other metabolic processes fundamental to plant growth (Cuciurean et al., 2024). However, extensive anthropogenic activities, including intensive agriculture, industrial effluents, improper waste disposal, and smelting operations, have markedly elevated soil metal concentrations above natural background levels across vast agricultural and peri-urban landscapes (Ali et al., 2013; Maitry et al., 2025b; Ramesh et al., 2024; Shah et al., 2022; Zerizghi et al., 2022).

Heavy metals, defined as elements with an atomic density >5 g cm^-^³ (Dalvi and Bhalerao, 2013; Laghlimi et al., 2015), present serious ecological concerns due to their toxicity, environmental persistence, and propensity for bioaccumulation and biomagnification across trophic levels (Manisalidis et al., 2020; Balraju et al., 2025). Among the most frequently detected are Pb, Cd, As, Hg, Cr, Zn, and Cu, several classified as United States Environmental Protection Agency (USEPA) priority pollutants due to their adverse effects on human and ecosystem health (Singh and Prasad, 2015). The prolonged half-lives of these elements in soils mean contamination events can have multigenerational consequences creating direct pathways for chronic health impacts, including organ damage, developmental disorders, and carcinogenesis (Tchounwou et al., 2012; Ahmad et al., 2024). The economic burden of metal contamination, from reduced crop yields to remediation costs, further underscores the urgency of developing effective and scalable restoration strategies.

While native vegetation in undisturbed ecosystems typically adapts to ambient trace metal concentrations, anthropogenic disturbances introduce non-native or metal-sensitive species to contaminated environments, triggering pronounced phytotoxic responses (Wuana and Okieimen, 2011; De Souza et al., 2023). Elevated metal concentrations induce physiological and metabolic disturbances in plants, including impaired photosynthesis, enzymatic disruption, and oxidative stress from reactive oxygen species (ROS) overproduction, ultimately reducing growth and productivity (Berni et al., 2019). Soil microorganisms are similarly affected, with metal-induced suppression of microbial activity disrupting essential biochemical processes such as nutrient cycling and organic matter decomposition (Patil and Faizan, 2017). Human-induced acidification from fertilizer overuse further exacerbates metal mobility and toxicity, amplifying these effects even without additional metal inputs (Fergus, 1954; Tchounwou et al., 2012).

Among remediation strategies, phytoremediation has emerged as an environmentally sustainable and economically viable approach for large-scale soil restoration (Muthusaravanan et al., 2018; Patil et al., 2023; Steliga and Kluk, 2020). This green technology harnesses plants’ natural abilities to accumulate, stabilize, or transform heavy metals through phytoextraction, phytostabilization, and rhizofiltration (Maitry et al., 2025c). Within this context, the genus *Eucalyptus* has attracted considerable research interest due to its rapid growth, high biomass production, deep root systems, and reported metal tolerance (Parmar and Singh, 2015; Sawalha et al., 2021; Shirazi et al., 2023). Several *Eucalyptus* species have shown strong performance under heavy metal contamination due to their efficient uptake, sequestration, and detoxification mechanisms. For example, *Eucalyptus camaldulensis* has been extensively studied for its capacity to accumulate Cd and zinc; recent work demonstrates enhanced tolerance through organic amendments that improve nutrient uptake and antioxidant defenses (Ouyang et al., 2024; Singh et al., 2020; Patil et al., 2023). *Eucalyptus tereticornis* exhibits promise in Pb-contaminated soils, often showing effective phytoextraction or stabilization (Singh et al., 2020). Research has also highlighted *Eucalyptus globulus* tolerance to Cu and As, including its ability to bioaccumulate metals in mine tailings (Reboredo et al., 2021). Recent field studies from abandoned gold mines in India confirm that *E. globulus* accumulates substantial amounts of gold (up to 6.14 mg kg^-^¹ in leaves), As, and Pb, demonstrating its exceptional potential for phytoremediation of multi-metal-contaminated sites (Arveti et al., 2025). Similarly, *Eucalyptus citriodora* demonstrates potential for multi-metal contaminated sites, with superior tolerance and root accumulation of Cd and Pb at early growth stages (Patil et al., 2023).

Despite several studies have evaluated heavy metal tolerance or accumulation in individual *Eucalyptus* species, direct comparative evaluations of multiple commercially important species under identical Cd and Pb stress conditions remain limited, particularly during the vulnerable seedling phase. Moreover, comparative information regarding growth responses, chlorophyll retention, phytotoxicity and tolerance indices under standardized experimental conditions is scarce. Therefore, the present study was undertaken to identify comparatively tolerant *Eucalyptus* species that may serve as potential candidates for future phytoremediation investigations.

It was hypothesized that (1) significant species-specific differences exist in Cd and Pb tolerance among the four *Eucalyptus* species; (2) Cd would exhibit greater phytotoxicity than Pb across all species; and (3) *Eucalyptus citriodora* would demonstrate superior tolerance characteristics that may support future phytoremediation applications compared to the other species based on growth performance, biomass allocation, and physiological responses.

To address this gap and test these hypotheses, the present study provides a comprehensive comparative evaluation of four *Eucalyptus* species, *E. tereticornis*, *E. camaldulensis*, *E. globulus*, and *E. citriodora*, identified from preliminary field surveys as superior early-stage performers under stressed conditions. To the best of the authors’ knowledge, this is the first side-by-side, multi-parameter assessment of these economically important species under identical controlled Cd and Pb stress conditions. By integrating morphological measurements, physiological indicators (photosynthetic efficiency, chlorophyll fluorescence), and multivariate statistical clustering, the present study provides a holistic comparative framework. Specifically, this study aimed to: (1) quantify growth and physiological responses to increasing Cd and Pb concentrations; (2) evaluate comparative growth performance, biomass allocation, chlorophyll retention and tolerance indices under Cd and Pb stress; and (3) identify the most promising candidate(s) for phytostabilization or phytoextraction based on integrated tolerance indices and metal partitioning coefficients. Understanding these species-specific responses is essential for developing evidence-based phytoremediation strategies applicable across diverse contaminated landscapes, from agricultural fields affected by fertilizer overuse to industrial sites burdened by legacy pollution, thereby advancing sustainable, nature-based solutions for soil restoration, environmental protection, and safeguarding human communities dependent on healthy soils.

## 2 Materials and methods

### 2.1 Study site, growing medium and experimental conditions

**Figure 1:**
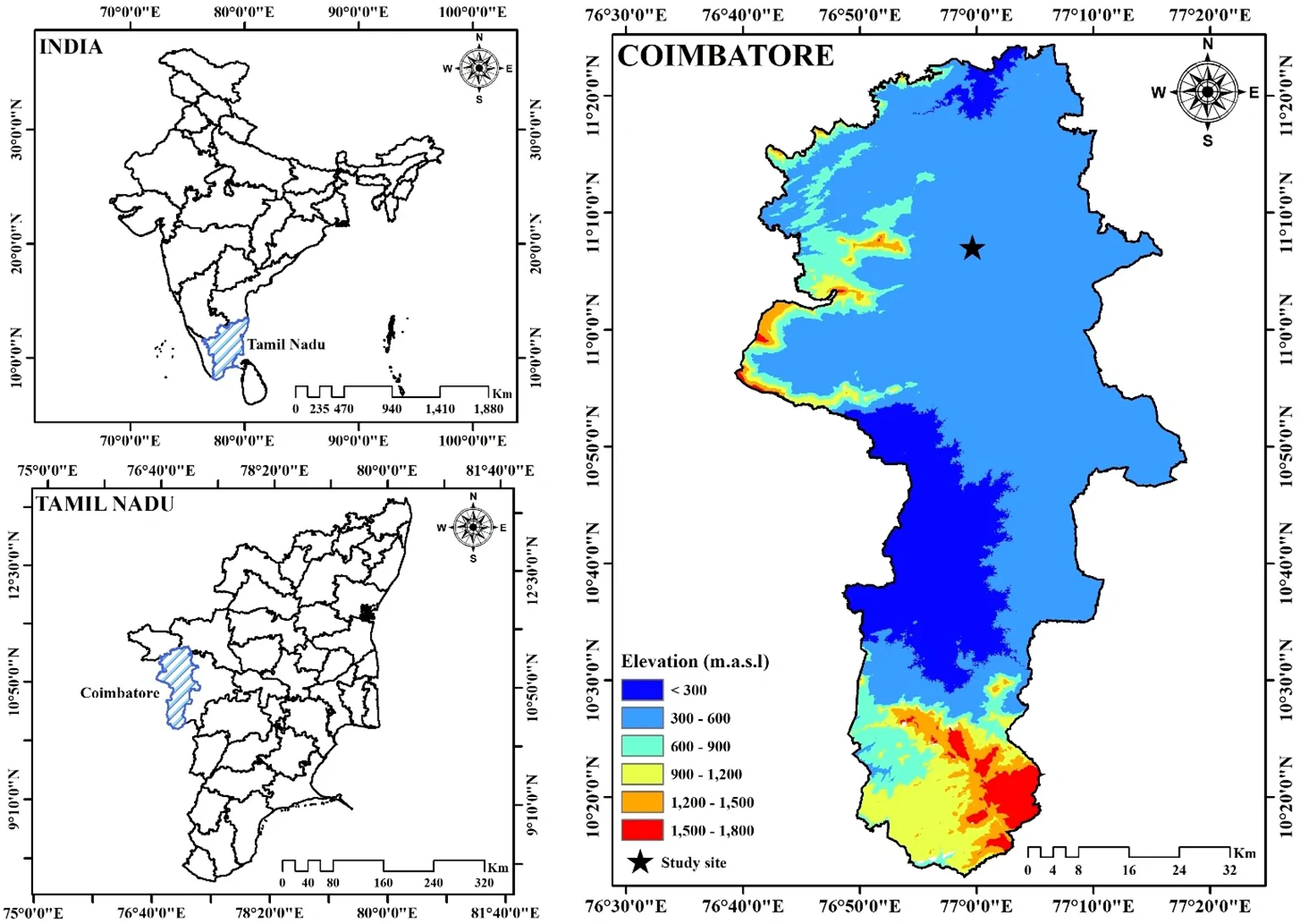
Geographic location of the study site. Map showing the position of Tamil Nadu Agricultural University, Coimbatore, India.

The experiment was conducted under controlled laboratory conditions at the Tamil Nadu Agricultural University, Coimbatore, India (**Figure 1**) (Latitude: 11° 07’ 3.36” N; Longitude: 76° 59’ 39.91” E), using sterilized red soil as the growing medium. Before metal treatment, the used growing medium (n=3) was slightly acidic (pH 6.54 ± 0.38) and non-saline (EC 0.09 ± 0.001 dS m^-^¹). It exhibited a lower field capacity (17.83 ± 0.71%), and permanent wilting point (8.07 ± 1.12%), indicating relatively poor moisture retention due to its coarse (sandy loam) texture. Although the soil contained moderate organic carbon (0.46 ± 0.07%), it showed higher available nitrogen (0.02 ± 0.0018%), phosphorus (0.03 ± 0.0014%), and potassium (0.02 ± 0.002%), suggesting comparatively better immediate nutrient availability (Maitry et al., 2025a). Controlled environmental conditions were maintained at 25 ± 1 °C temperature, 65–70% relative humidity, and a 16 h photoperiod with a photosynthetic photon flux density of approximately 250 μmol m^-^² s^-^¹.

### 2.2 Species selection and rationale

Based on the results of multiple literature reviews, four *Eucalyptus* species: *E. tereticornis*, *E. camaldulensis*, *E. globulus*, and *E. citriodora*, exhibiting superior early-stage growth under stressed conditions, were selected for detailed investigation. These species have been documented in the literature as strong performers under heavy-metal contamination due to their efficient uptake, sequestration, and detoxification abilities (Bilal et al., 2014; Maiti and Rana, 2017).

### 2.3 Experimental design and treatment details

A factorial completely randomized design (CRD) was employed with four *Eucalyptus* species and seven treatment levels; each replicated three times. Cd and Pb were selected as the test metals based on their environmental prevalence, high toxicity to plants, and relevance to phytoremediation research. Both metals are classified as priority pollutants by the United States Environmental Protection Agency (USEPA) and are frequently detected together in contaminated soils due to shared anthropogenic sources such as industrial emissions, fertilizer application, and atmospheric deposition (Nagajyoti et al., 2010; Tchounwou et al., 2012). Cd is readily taken up by plants and induces chlorosis, growth inhibition, and oxidative stress even at low concentrations, while Pb, though less mobile in soils, accumulates in roots and disrupts fundamental physiological processes, including photosynthesis and cell division (Pourrut et al., 2011; Singh and Prasad, 2015). The co-occurrence of Cd and Pb in many contaminated landscapes, from agricultural fields to industrial sites, makes understanding plant responses to these metals critical for developing effective phytoremediation strategies (Wuana and Okieimen, 2011). Analytical reagent (AR) grade salts, CdCl_2_ and Pb (NO_3_)_2_, served as the sources of Cd and Pb, respectively (Patil and Umadevi, 2014). The concentrations of Cd and Pb were selected based on established critical limits (Chen and Lee, 1997; Patil and Umadevi, 2014) and included levels below, at, and above the critical point representing moderate to highly contaminated conditions, thereby simulating stressed remediation scenarios (**Table 1**). The metal salts were mixed thoroughly with sterilized red soil before sowing to ensure uniform distribution. A complete description of the treatment combinations used in the study is presented in **Table 2**.

**Table 1:** Concentration levels of heavy metals used in the investigation.

S. no.	Heavy metals	Salts used	Concentration levels (mg kg^-1^)		
			*Below critical point*	*Critical*	*Above critical point*
1.	M1-Cd (Cd)	CdCl_2_	25	50	100
2.	M2-Pb (Pb)	Pb(NO_3_)_2_	100	125	250

**Table 2:** Treatment combinations used in the investigation.

Content	Details
Plant species (S)	S1 - *Eucalyptus tereticornis* S2 - *E. camaldulensis* S3 - *E. globulus* S4 - *E. citriodora*
Treatments (T)	T_0_ – Control (No metal) T1 - Cd 25 mg kg^-1^ T2 - Cd 50 mg kg^-1^ T3 - Cd 100 mg kg^-1^ T4 - Pb 100 mg kg^-1^ T5 - Pb 125 mg kg^-1^ T6 - Pb 250 mg kg^-1^
Replications	3
Design	Factorial Completely Randomized Design (CRD)

Values are mean ± S.E. (n = 3). Different letters in the same column indicate a significant difference at p<0.05 (Duncan’s Multiple Range Test).

### 2.4 Screening and seed germination

For each species, 25 seeds were sown per replication in plastic bowls containing 250 g of sterilized red soil amended with the predetermined metal concentrations. Seeds germinated within approximately 7–10 days after sowing. After establishment, seedlings were thinned to ten healthy and uniform seedlings per bowl at the two-leaf stage to maintain consistency. To ensure continuous metal exposure throughout the experiment, cadmium (Cd) and lead (Pb) solutions at the respective treatment concentrations (**Table 2**) were prepared in 45 mL of distilled water and applied to each plastic bowl at 30-day intervals over the 120-day experimental period. The required Cd and Pb concentrations were slightly adjusted using the appropriate correction factors before application, considering the density of water at 25 °C (0.9970 g mL^-^¹; PubChem CID: Pure Water Density Standard, UKAS ISO/IEC 17025 and ISO Guide 34 certified). The solution volume was determined based on the field capacity of the sandy loam soil (17.83%) and the dry soil weight (250 g), following the approach described by Ratliff et al. (1983), which yielded a water requirement of approximately 44.6 mL (250 g × 17.83%). This value was rounded to 45 mL to maintain soil moisture at field capacity while preventing excess water and leachate formation.

### 2.5 Growth parameter measurements

After 120 days of treatment, three plants per replication were randomly selected and harvested for growth parameter analysis. The following parameters were recorded:

1. Shoot length (cm)
2. Root length (cm)
3. Collar diameter (mm)
4. Shoot dry weight (g plant^-^¹)
5. Root dry weight (g plant^-^¹)
6. Total dry matter (g plant^-^¹)
7. Shoot-to-root ratio

Dry weights were recorded after oven-drying at 70 °C until a constant weight was achieved.

Phytotoxicity of root and shoot was determined based on the studied plant growth parameters by using the following formula (Turner and Marshall, 1972):


$$Phytotoxicity\;(\%)=\frac{Root\;or\;Shoot\;length\;of\;treated\;plant-Root\;or\;Shoot\;length\;of\;control}{Root\;or\;Shoot\;length\;of\;control}\;\times 100$$

The tolerance index (TI) was calculated following Wilkins (1978):


$$TI(\%)=\frac{Total\;dry\;matter\;of\;treated\;plant}{Total\;dry\;matter\;of\;control}\times 100$$

### 2.6 Total chlorophyll content analysis

Total chlorophyll content was estimated using the method of Arnon (1949) using fully expanded fresh leaves collected from three randomly selected seedlings within each replication. The fresh leaf samples were then homogenized in a mortar and pestle with 10 mL of 80% acetone and kept overnight at 4 °C for complete pigment extraction. The extract was centrifuged at 2500 rpm for 20 minutes, and the supernatant was collected and diluted with cold 80% acetone to a final volume of 15 mL. Absorbance was measured at 645 nm and 663 nm using a spectrophotometer, with 80% acetone as the blank.

Total chlorophyll content was calculated using the formula:


$$Total\;Chlorophyll\;(mg\;g^{-1}\;FW)\;=\frac{\left[(8.02\times A_{663})+(20.2\;\times A_{645})\right]}{1000\;\times W}\times V$$

Where: $A_{663}$ and $A_{645}$ = absorbance at respective wavelengths; V = final volume of solution (15 mL); W = fresh weight of leaf sample (g).

### 2.7 Statistical analysis

All data were compiled and tabulated using Microsoft Excel. Analysis of variance (ANOVA) for the factorial completely randomized design was performed using MS-DOS AGDATA/AGRES software. Mean comparisons were conducted using Duncan’s Multiple Range Test (DMRT) at P≤0.05 significance level with SPSS software (Version 25.0, IBM Corp., USA). To visualize multivariate relationships among species, treatments, and physiological responses, a scatterplot matrix and hierarchical cluster analysis (HCA) were performed using SPSS (Version 25.0). Heatmap clustering and correlation matrices were generated using OriginPro 2025b (OriginLab Corp., USA).

## 3 Results

The comprehensive assessment of four *Eucalyptus* species under controlled Cd and Pb stress revealed significant species-specific differences in growth performance, biomass allocation, and physiological responses. While metal exposure generally suppressed plant development across all species, the magnitude of inhibition varied markedly depending on metal type, concentration, and inherent species tolerance. *E. citriodora* consistently outperformed the other species, maintaining superior growth and chlorophyll content even at the highest metal concentrations, whereas *E. camaldulensis* exhibited the greatest sensitivity, particularly to Cd. The results presented below elucidate the differential responses of these species and provide a quantitative basis for selecting optimal candidate species for future phytoremediation studies in metal-contaminated soils.

### 3.1 Shoot and root length

**Figure 2:**
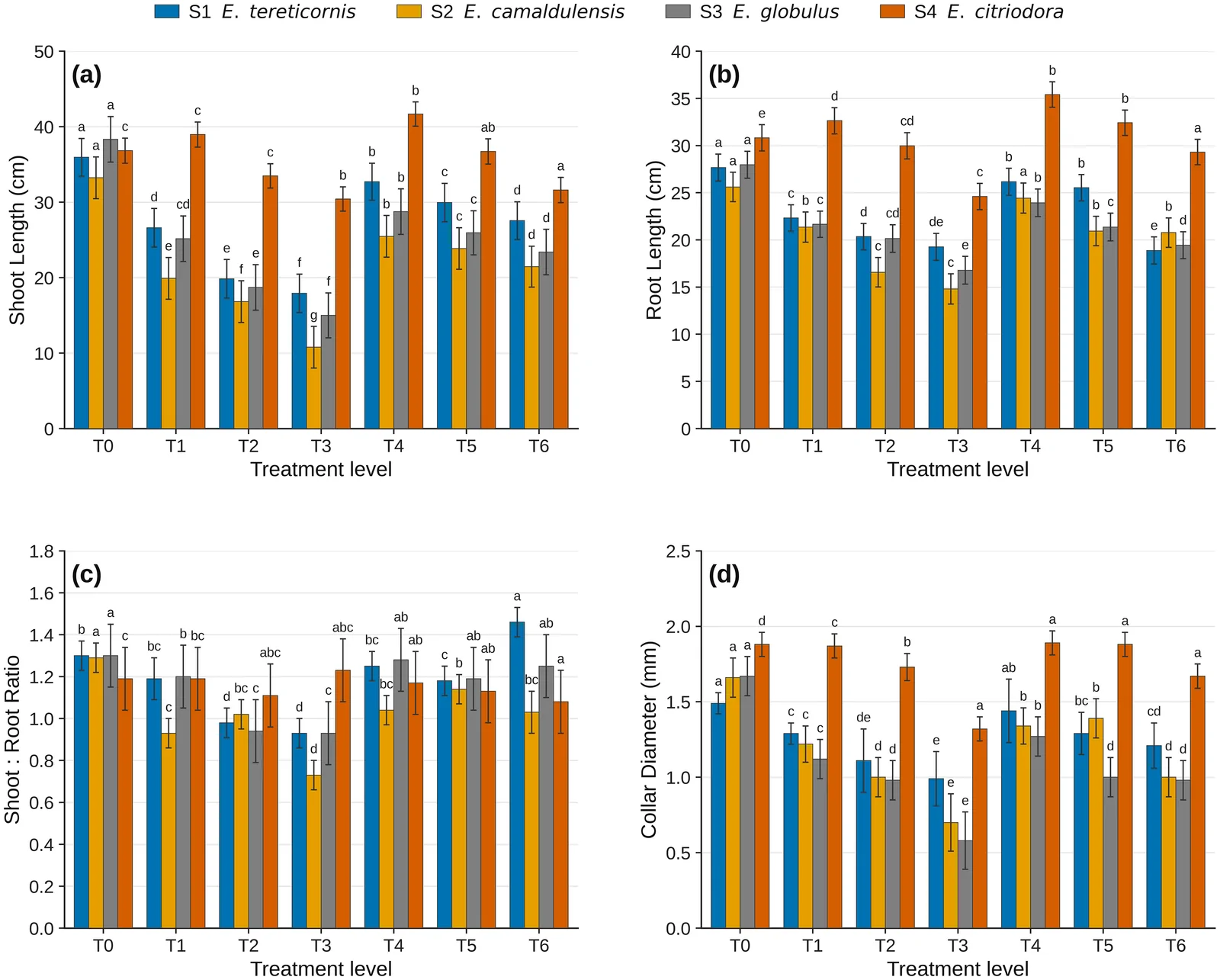
**(a)** Shoot length (cm); **(b)** root length (cm); **(c)** shoot: root ratio; **(d)** collar diameter (mm) of different *Eucalyptus* spp. under treatment with Pb and Cd at different levels. The data points and error bars represent the means and standard error of three replicates (n = 3), with different letters indicating significant differences (p<0.05) between treatments based on Duncan’s Multiple Range Test.

Pb applied at 100 mg kg^-^¹, and Cd at 25 mg kg^-^¹, increased shoot length in *E. citriodora*, reaching 41.67 cm and 39.00 cm, respectively, compared with the control and other treatments. The greatest reduction in shoot length occurred under the highest Cd concentration (100 mg kg^-^¹), where shoot lengths declined to 17.93 cm, 10.83 cm, 15.07 cm, and 30.43 cm in *E. tereticornis*, *E. camaldulensis*, *E. globulus*, and *E. citriodora*, respectively (**Figure 2**). All other treatments exhibited a notable decline in shoot length compared with the controls across the evaluated species.

A similar trend was observed for root length. Pb at 100 mg kg^-^¹ and 125 mg kg^-^¹, and Cd at 25 mg kg^-^¹, enhanced root length in *E. citriodora*, producing lengths of 35.43 cm, 32.43 cm, and 32.67 cm, respectively. Cd at 100 mg kg^-^¹ resulted in the lowest root lengths of 19.30 cm, 14.90 cm, 16.80 cm, and 24.67 cm in *E. tereticornis*, *E. camaldulensis*, *E. globulus*, and *E. citriodora*, respectively (**Figure 2**). *E. tereticornis*, *E. camaldulensis*, and *E. globulus* exhibited their greatest shoot and root growth under control conditions.

### 3.2 Shoot to root ratio

The highest shoot: root ratio was recorded in *E. tereticornis* under Pb treatment at 250 mg kg^-^¹, reaching 1.46, whereas the lowest ratio (0.73) was observed in *E. camaldulensis* under Cd treatment at 100 mg kg^-^¹ (**Figure 2**). Cd at 100 mg kg^-^¹ also resulted in the lowest shoot: root ratio for *E. globulus*, while Pb at 250 mg kg^-^¹ caused the lowest ratio in *E. citriodora*. The highest shoot: root ratio for *E. camaldulensis* and *E. globulus* was recorded under control conditions (1.30), while *E. citriodora* displayed its highest ratio (1.23) under Cd treatment at 100 mg kg^-^¹.

### 3.3 Collar diameter

Among the studied species, *E. citriodora* consistently exhibited the highest collar diameter across all treatment levels (**Figure 2**). The maximum collar diameter for this species (1.89 mm) was recorded under Pb treatment at 100 mg kg^-^¹. In contrast, *E. tereticornis*, *E. camaldulensis*, and *E. globulus* showed their best collar diameter growth under control conditions, with values of 1.48 mm, 1.66 mm, and 1.67 mm, respectively. The lowest collar diameters overall were observed in *E. globulus* under various Pb and Cd treatments. Species-wise comparison revealed that the minimum collar diameter for all four species occurred under Cd treatment at 100 mg kg^-^¹, recording 0.99 mm, 0.70 mm, 0.58 mm, and 1.32 mm for *E. tereticornis*, *E. camaldulensis*, *E. globulus*, and *E. citriodora*, respectively.

### 3.4 Dry biomass of shoot and root

**Figure 3:**
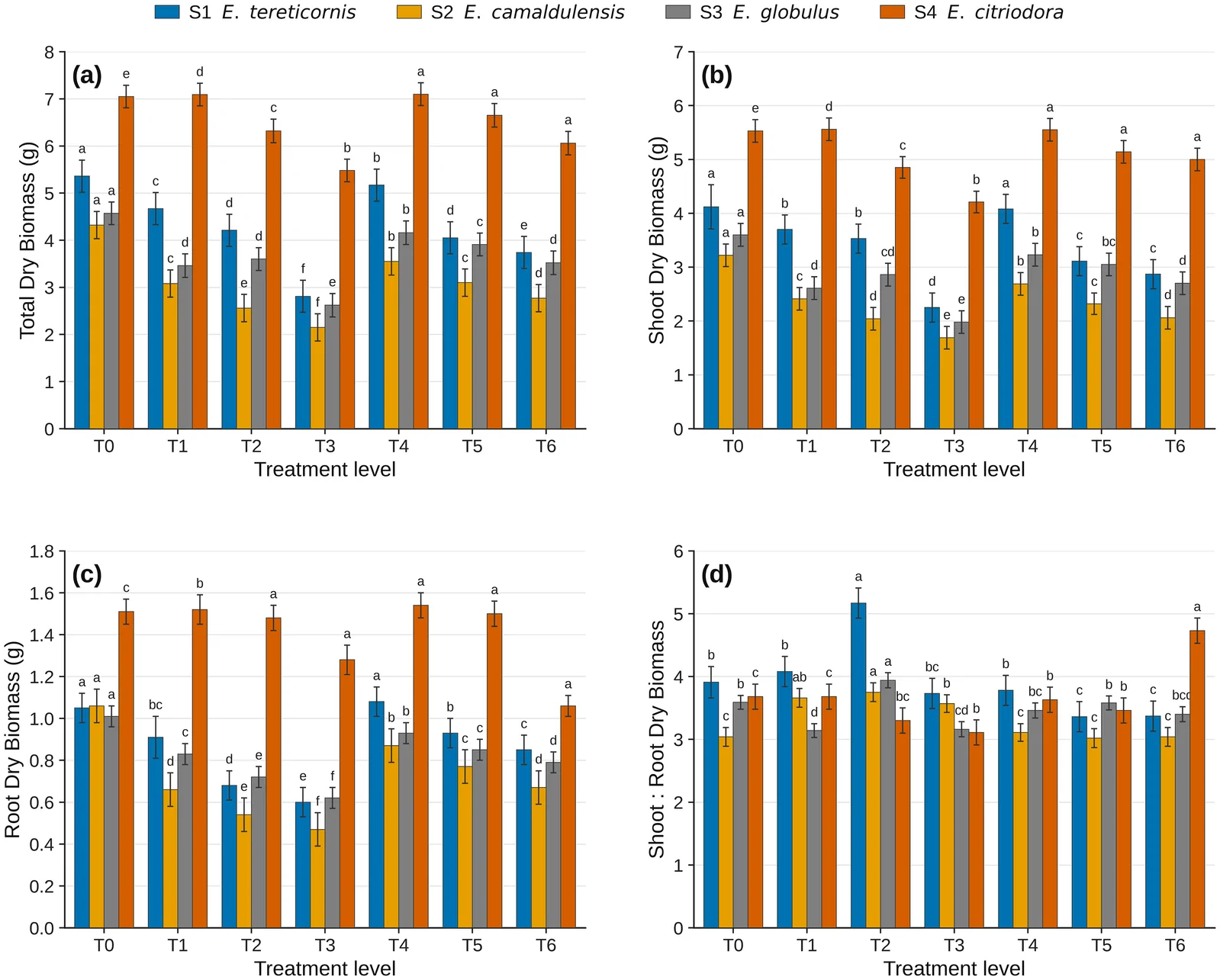
**(a)** Total dry biomass (g); **(b)** shoot dry biomass (g); **(c)** root dry biomass (g); **(d)** shoot: root dry biomass of different *Eucalyptus* spp. under treatment with Pb and Cd at different levels. The data points and error bars represent the means and standard error of three replicates (n = 3), with different letters indicating significant differences (p<0.05) between treatments based on Duncan’s Multiple Range Test.

The application of heavy metals significantly influenced the dry weights of shoots and roots. *E. citriodora* produced the highest total dry biomass across all Pb and Cd treatments (**Figure 3**). The maximum total dry biomass (7.10 g) was recorded in *E. citriodora* under Pb treatment at 100 mg kg^-^¹. The highest root dry biomass in this species was 1.54 g, while shoot dry biomass peaked at 5.57 g under Cd treatment at 25 mg kg^-^¹, followed closely by 5.55 g under Pb at 100 mg kg^-^¹ (**Figure 3**).

*E. tereticornis* exhibited its highest root dry biomass under Pb at 100 mg kg^-^¹ (1.09 g), followed by the control (1.06 g) (**Figure 3**). A marked decline in shoot and root dry biomass was observed in all species under Cd at 100 mg kg^-^¹, resulting in reduced total dry biomass for *E. tereticornis*, *E. camaldulensis*, *E. globulus*, and *E. citriodora* at 2.81 g, 2.18 g, 2.62 g, and 5.49 g, respectively. Among these, *E. camaldulensis* displayed the lowest total dry biomass (2.18 g).

### 3.5 Shoot to root dry biomass ratio

The highest shoot-to-root dry biomass ratio was observed in *E. tereticornis* under Cd treatment at 50 mg kg^-^¹, reaching 5.14, whereas the lowest ratio was recorded in *E. camaldulensis* under Pb treatment at 125 mg kg^-^¹, with a value of 3.00 (**Figure 3**). For *E. globulus* and *E. citriodora*, the highest shoot-to-root dry biomass ratios were 3.56 under control conditions and 4.70 under Pb treatment at 125 mg kg^-^¹, respectively. The lowest ratios for *E. globulus* and *E. citriodora* were observed under Cd treatments at various concentrations, whereas for *E. tereticornis* and *E. camaldulensis*, the lowest values occurred under different Pb treatments.

### 3.6 Phytotoxicity of shoot and root

Phytotoxicity in both shoots and roots of all four *Eucalyptus* species increased with rising concentrations of heavy metals and decreased at lower concentrations. Roots generally exhibited higher phytotoxicity than shoots (**Tables 3** and **4**).

**Table 3:** Phytotoxicity of the shoots of the studied *Eucalyptus* species under different treatments.

Treatment	*S1*	*S2*	*S3*	*S4*
*T1M1*	25.91 ± 0.84^c^	48.00 ±1.10^c^	27.86 ±0.26^cd^	-8.41 ±5.65^bc^
*T2M1*	44.66 ± 1.07^b^	57.21 ±0.10^b^	44.70 ±0.31^b^	6.51 ±0.78^a^
*T3M1*	50.05 ± 0.89^a^	75.35 ±1.46^a^	54.25 ±3.67^a^	14.83 ±0.63^a^
*T4M2*	9.01 ± 2.99^e^	31.26 ±0.76^f^	18.32 ±0.83^e^	-15.64 ±3.26^c^
*T5M2*	16.53 ± 0.79^d^	36.07 ±0.80^e^	25.87 ±1.26^d^	-2.26 ±1.73^b^
*T6M2*	23.31 ±1.73^c^	43.39 ±1.12^d^	32.38 ±1.66^c^	11.57 ±0.86^a^

Values are mean ± S.E. (n = 3). Different letters in the same column indicate a significant difference at p<0.05 (Duncan’s Multiple Range Test).

**Table 4:** Phytotoxicity of the root of the studied *Eucalyptus* species under different treatments.

Treatment	*S1*	*S2*	*S3*	*S4*
*T1M1*	49.04 ±1.20^c^	56.36 ±5.82^bc^	50.83 ±2.38^d^	10.49 ±1.08^c^
*T2M1*	56.27 ±1.20^b^	75.06 ±4.22^a^	56.19 ±0.60^bc^	19.14 ±0.00^b^
*T3M1*	60.00 ±1.63^ab^	81.82 ±2.15^a^	68.21 ±0.21^a^	36.43 ±1.08^a^
*T4M2*	35.18 ±0.60^d^	44.29 ±5.25^c^	42.50 ±2.06^e^	1.51 ±1.13^d^
*T5M2*	37.47 ±2.31^d^	58.05 ±2.25^b^	51.67 ±0.78^cd^	11.24 ±0.71^c^
*T6M2*	61.57 ±2.14^a^	58.70 ±1.89^b^	58.69 ±1.45^b^	21.30 ±2.16^b^

Values are mean ± S.E. (n = 3). Different letters in the same column indicate a significant difference at p<0.05 (Duncan’s Multiple Range Test).

Among the species, *E. camaldulensis* showed the highest shoot phytotoxicity under both Cd and Pb treatments, with values of 75.35 ± 1.46 and 43.39 ± 1.12, respectively. It was followed by *E. globulus* (54.25 ± 3.67 and 32.38 ± 1.66) and *E. tereticornis* (50.05 ± 0.89 and 23.31 ± 1.73). The lowest phytotoxicity levels were observed in *E. citriodora*, with values of −8.41 ± 5.65 and −15.64 ± 3.26 under the lowest Cd (25 mg kg^-^¹) and Pb (100 mg kg^-^¹) treatments, respectively.

In roots, *E. camaldulensis* exhibited the highest phytotoxicity under Cd treatment, with a value of 81.82 ± 2.15, followed by *E. globulus* (68.21 ± 0.21) and *E. tereticornis* (60.00 ± 1.63). Under Pb treatment, the highest root phytotoxicity was observed in *E. tereticornis* at 250 mg kg^-^¹, reaching 61.57 ± 2.14. Following *E. tereticornis*, *E. camaldulensis*, and *E. globulus* exhibited near-equivalent root phytotoxicity levels of 58.70 ± 1.89 and 58.69 ± 1.45, respectively. The lowest root phytotoxicity levels for both metals were recorded in *E. citriodora*, with values of 10.49 ± 1.08 and 1.51 ± 1.13 under low-level treatments.

### 3.7 Tolerance index

The Tolerance Index (TI) decreased significantly with increasing heavy metal concentrations and increased at lower concentrations (**Table 5**). Among the studied species, *E. citriodora* consistently exhibited the highest tolerance index across all treatment levels, followed by *E. globulus*. Under lower treatments of Cd at 25 mg kg^-^¹ and Pb at 100 mg kg^-^¹, *E. tereticornis* displayed higher tolerance indices (87.04 ± 0.93 and 96.39 ± 1.40, respectively) compared to *E. globulus* (75.73 ± 0.15 and 91.16 ± 0.53, respectively). *E. globulus* showed its highest TI under Cd treatment at 50 mg kg^-^¹ (78.86 ± 0.15). *E. camaldulensis* exhibited the lowest tolerance index across all concentrations of both metals. Overall, tolerance indices were higher under Pb treatments compared to Cd, with the maximum TI observed at Pb 100 mg kg^-^¹ and the minimum at Cd 100 mg kg^-^¹.

**Table 5:** Tolerance index of studied *Eucalyptus* species under different treatments.

Treatment	*S1*	*S2*	*S3*	*S4*
*T1M1*	87.04 ±0.93^b^	71.60 ±0.23^b^	75.73 ±0.15^d^	100.63 ±0.02^a^
*T2M1*	78.66 ±0.14^c^	59.39 ±0.43^d^	78.86 ±0.15^c^	89.72 ±0.11^c^
*T3M1*	52.48 ±0.91^f^	50.32 ±0.36^e^	57.39 ±0.34^e^	77.86 ±0.47^e^
*T4M2*	96.39 ±1.40^a^	82.42 ±0.43^a^	91.16 ±0.53^a^	100.73 ±0.46^a^
*T5M2*	75.51 ±0.33^d^	71.96 ±0.34^b^	85.69 ±0.76^b^	94.45 ±0.41^b^
*T6M2*	69.77 ±0.41^e^	64.23 ±0.34^c^	77.16 ±0.89^d^	86.09 ±0.47^d^

Values are mean ± S.E. (n = 3). Different letters in the same column indicate a significant difference at p<0.05 (Duncan’s Multiple Range Test).

### 3.8 Correlation analysis and hierarchical clustering

**Figure 4:**
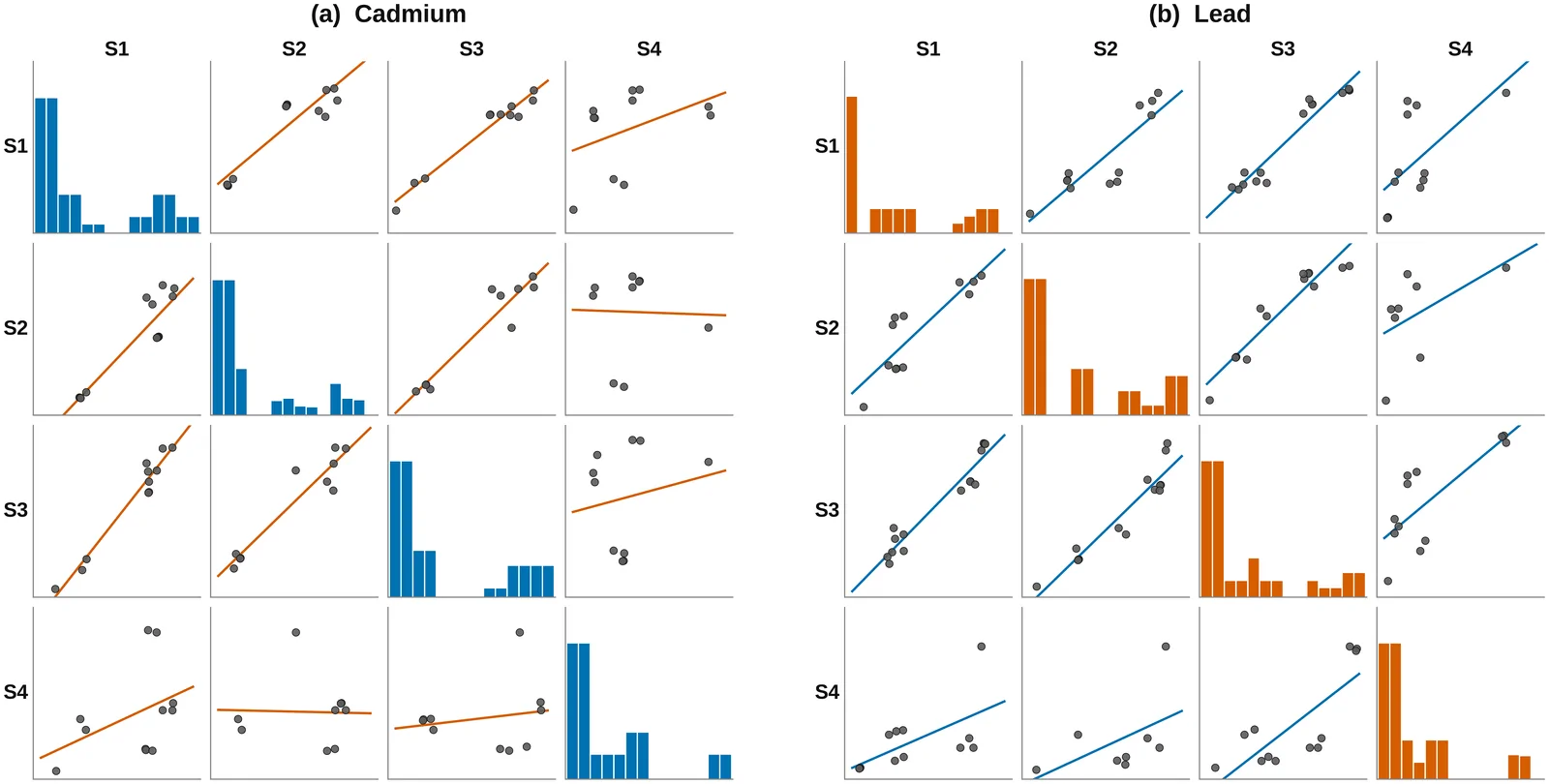
Scatterplot matrix of correlation between different studied parameters of *Eucalyptus* species: shoot length, root length, shoot: root ratio, collar diameter, total dry biomass, shoot dry biomass, root dry biomass, shoot: root dry biomass, phytotoxicity of shoot, phytotoxicity of root, and tolerance index for **(a)** Cd and **(b)** Pb heavy metal.

Statistical analysis revealed significant linear correlations among multiple growth parameters at treatment levels exceeding critical points (100 mg kg^-^¹ for Cd and 250 mg kg^-^¹ for Pb). All species exhibited significant positive correlations among parameters (**Figure 4**). The strongest positive correlations were observed between *E. tereticornis* and *E. globulus*, with correlation coefficients of 0.994 and 0.988 under Cd and Pb treated soils, respectively, at p < 0.01 (2-tailed). Variables related to shoot and root phytotoxicity displayed relatively weaker correlations.

**Figure 5:**
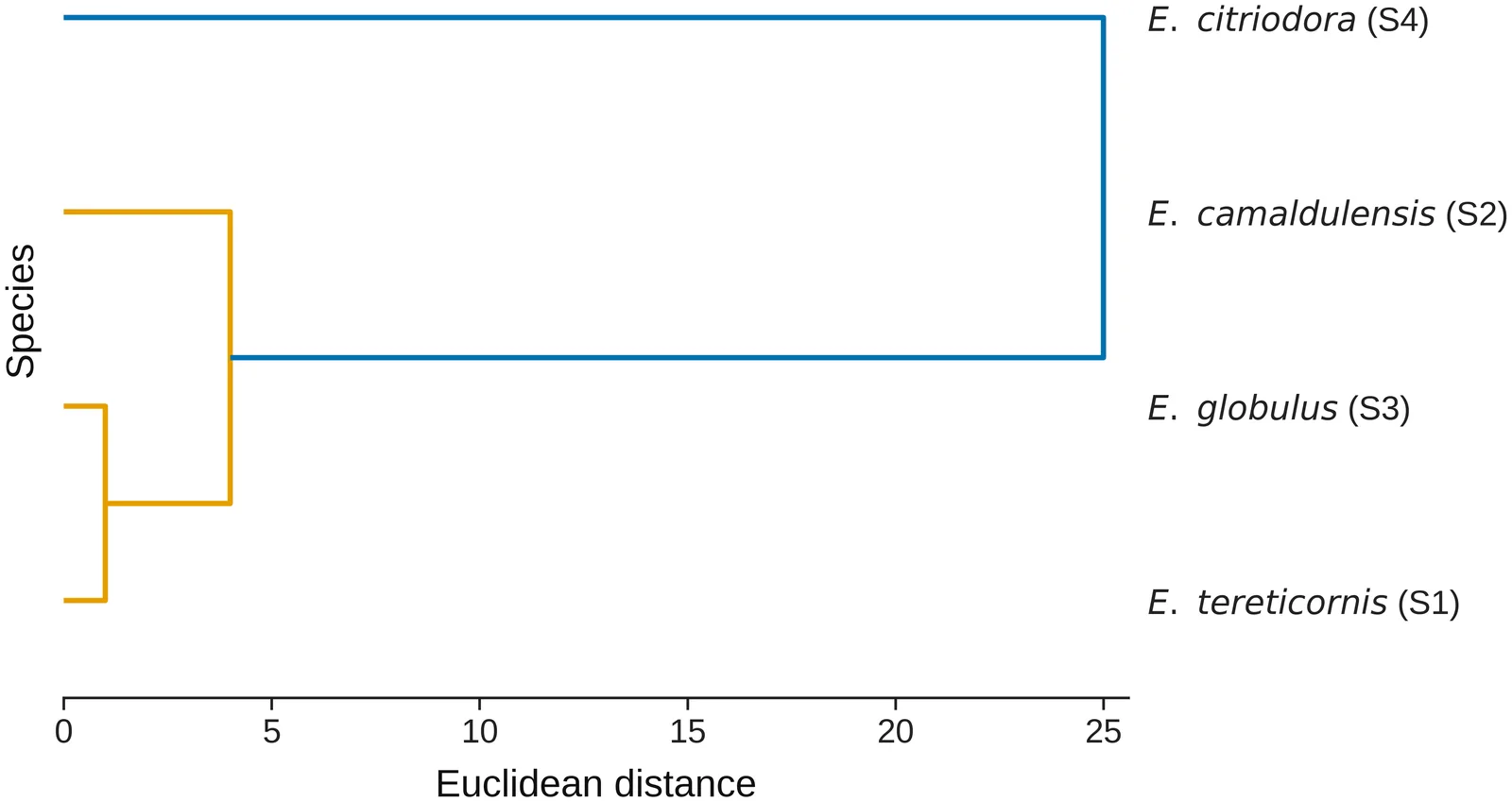
Agglomerative hierarchical clustering of studied *Eucalyptus* species, *E. tereticornis*, *E. camaldulensis*, *E. globulus*, and *E. citriodora*, showing similarities and dissimilarities among the species.

Agglomerative hierarchical clustering classified the four species based on their capacity to tolerate and accumulate heavy metals. *E. tereticornis* (S1) and *E. globulus* (S3) clustered closely together. *E. camaldulensis* (S2) formed a separate cluster closer to *E. tereticornis*. *E. citriodora* (S4) formed an independent cluster, demonstrating superior growth and tolerance under all treatment conditions (**Figure 5**). Based on hierarchical clustering, the species can be sequenced according to growth performance and phytoremediation potential as: S4 (*E. citriodora*) > S2 (*E. camaldulensis*) > S1 (*E. tereticornis*) > S3 (*E. globulus*).

### 3.9 Total chlorophyll content

Under control conditions, baseline chlorophyll content ranged from 1.75–1.92 mg g^-^¹ fresh weight, with *E. globulus* showing the highest baseline values. Cd exposure induced progressive chlorophyll degradation in a concentration-dependent manner. At 25 mg kg^-^¹ Cd, *E. camaldulensis* and *E. globulus* declined by approximately 31–33%, while *E. Citriodora* showed only 0.53% reduction (**Table 6**). At 50 mg kg^-^¹ Cd, reductions intensified, with *E. camaldulensis* experiencing a 47.43% decline compared to *E. citriodora*’s 11.17% reduction. At the highest Cd concentration (100 mg kg^-^¹), *E. camaldulensis* was most severely affected (64.57% reduction), followed by *E. globulus* (55.21%), *E. tereticornis* (44.02%), and *E. citriodora* (25.00%).

**Table 6:** Total chlorophyll content (mg g^-^¹ FW) of studied *Eucalyptus* species under different treatments.

Treatment	*S1*	*S2*	*S3*	*S4*
*T0*	1.84 ± 0.012^ab^	1.75 ± 0.04^b^	1.92 ± 0.099^a^	1.88 ± 0.017^a^
*T1M1*	1.65 ± 0.014^b^	1.2 ± 0.017^d^	1.28 ± 0.019^c^	1.89 ± 0.029^a^
*T2M1*	1.19 ± 0.019^b^	0.92 ± 0.027^c^	1.2 ± 0.023^b^	1.67 ± 0.01^a^
*T3M1*	1.03 ± 0.023^b^	0.62 ± 0.012^d^	0.86 ± 0.018^c^	1.41 ± 0.017^a^
*T4M2*	2.06 ± 0.194^a^	1.3 ± 0.011^c^	1.5 ± 0.015^b^	1.92 ± 0.025^a^
*T5M2*	1.51 ± 0.017^b^	1.04 ± 0.031^d^	1.36 ± 0.016^c^	1.61 ± 0.01^a^
*T6M2*	1.34 ± 0.016^a^	0.83 ± 0.026^b^	1.01 ± 0.009^b^	1.41 ± 0.272^a^

Values are mean ± S.E. (n = 3). Different letters in the same column indicate a significant difference at p<0.05 (Duncan’s Multiple Range Test).

Pb stress exhibited a biphasic response pattern. At 100 mg kg^-^¹ Pb, *E. tereticornis* showed an 11.96% increase in chlorophyll content (reaching 2.06 ± 0.194 mg g^-^¹), and *E. citriodora* increased by 2.13%. At higher Pb concentrations (125 and 250 mg kg^-^¹), all species displayed dose-dependent declines, with *E. camaldulensis* most affected (40.57% and 52.57% reductions) and *E. citriodora* most resistant (14.36% and 25.00% reductions).

Cd consistently produced more severe chlorophyll reductions than Pb across all species and concentrations. Average chlorophyll reductions across Cd treatments were 29.9% (*E. tereticornis*), 47.8% (*E. camaldulensis*), 42.0% (*E. globulus*), and 11.9% (*E. citriodora*), compared to 11.1%, 39.6%, 32.8%, and 12.4% for Pb treatments, respectively (**Table 6**).

**Figure 6:**
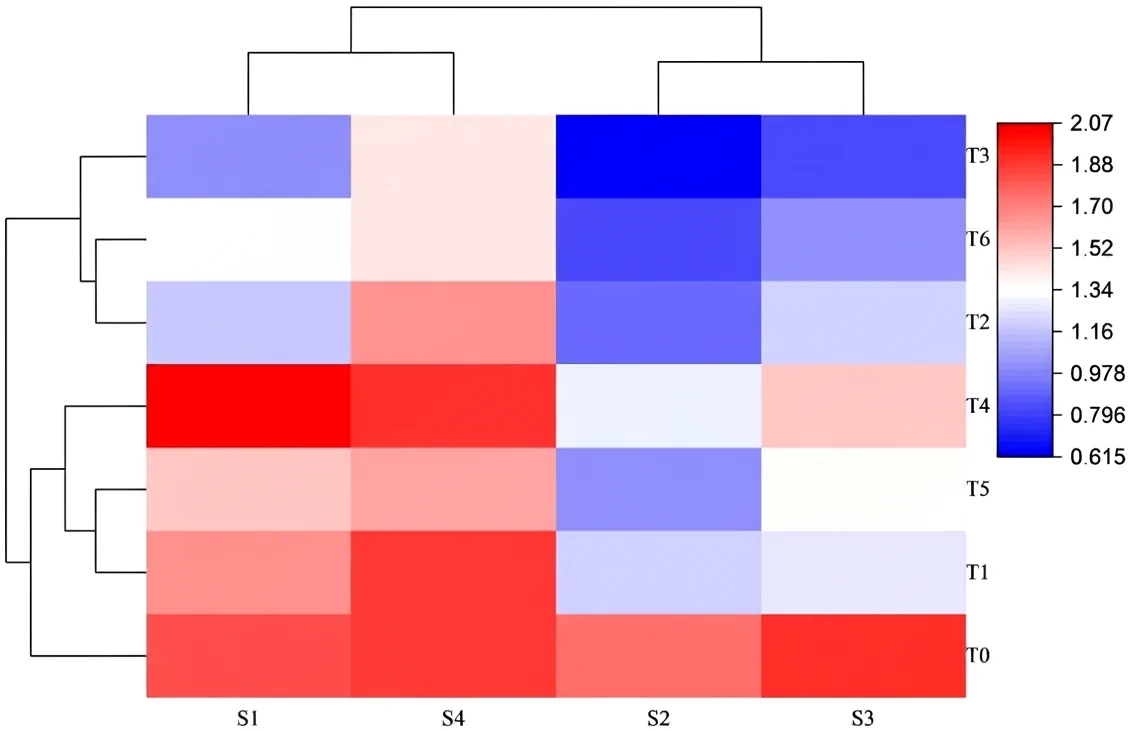
Heatmap clustering of total chlorophyll content among four *Eucalyptus* species (S1–S4) under different heavy-metal treatments and control (T_0_–T_6_).

Heatmap clustering illustrated variation in total chlorophyll content (**Figure 6**). *E. citriodora* (S4) and *E. tereticornis* (S1) maintained comparatively higher chlorophyll across most treatments. *E. camaldulensis* (S2) and *E. globulus* (S3) showed greater chlorophyll reduction under increasing Cd (T2, T3) and Pb (T6) stress.

### 3.10 Correlation of total chlorophyll with growth parameters

**Figure 7:**
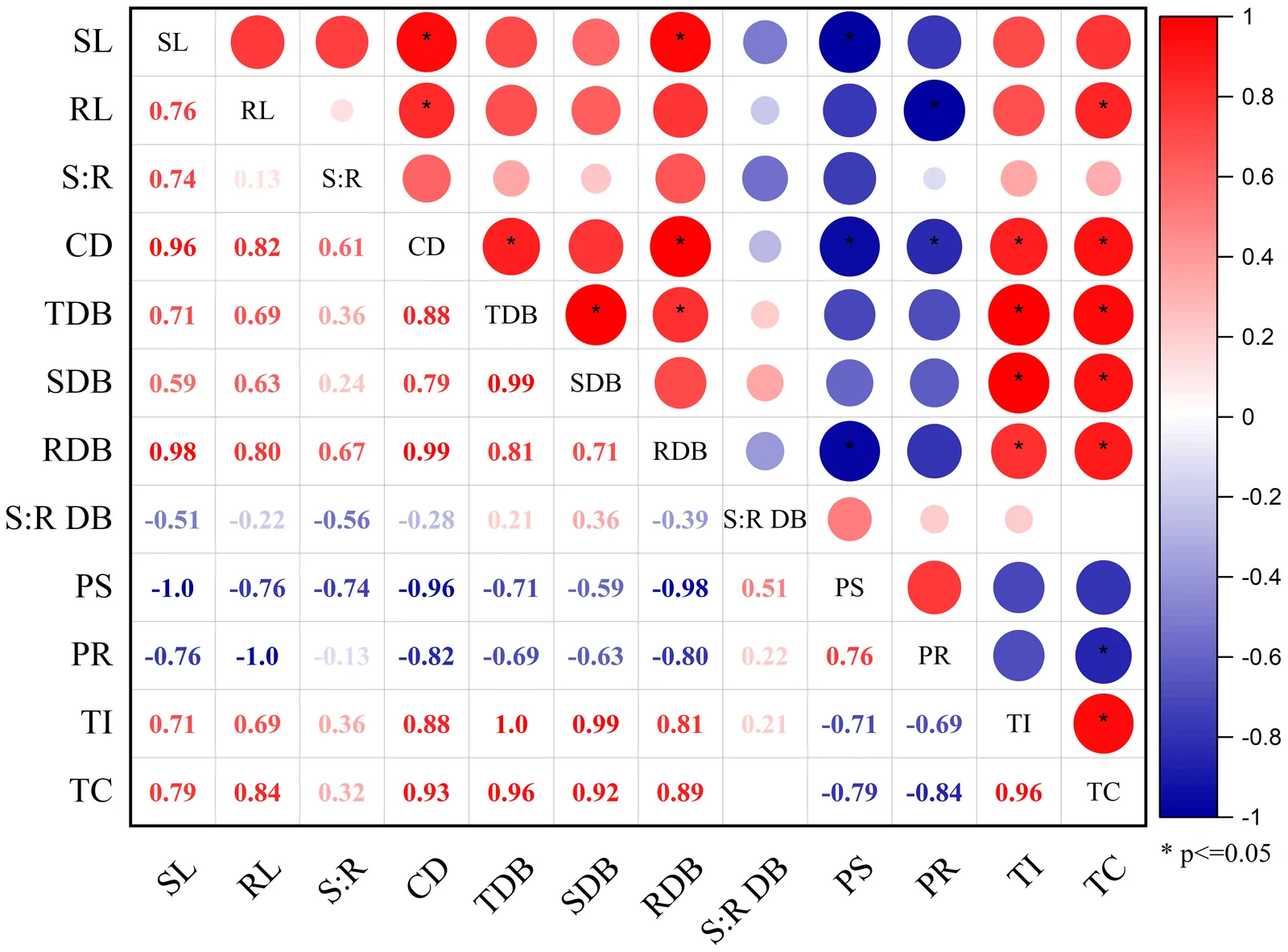
Correlation analysis (significant level: 0.05) of total chlorophyll content with various studied growth parameters among four Eucalyptus species under different heavy-metal treatments and control. Here, SL is shoot length; RL is root length; S:R is shoot root ratio; CD is collar diameter; TDB Total dry biomass; SDB is shoot dry biomass; RDB is root dry biomass, S:R DB shoot root dry biomass ratio; PS phytotoxicity of shoot; PR is phytotoxicity of root; TI is tolerance index and TC is total chlorophyll *p<=0.05.

Correlation analysis indicated that total chlorophyll (TC) maintained strong positive relationships with major growth parameters (**Figure 7**). TC showed very high correlations with total dry biomass, shoot dry biomass, root dry biomass, and collar diameter. Moderate positive correlations were observed between TC and shoot length, root length, and shoot–root ratio. TC was strongly negatively correlated with shoot and root phytotoxicity.

## 4 Discussion

### 4.1 Differential toxicity of Cd and Pb

The results consistently demonstrated that Cd exhibited significantly greater phytotoxicity than Pb across all growth parameters and species. This differential toxicity can be attributed to several mechanisms. Cd is readily taken up by plants and interferes with photosynthetic processes, nutrient uptake, and enzyme activity (Kabata-Pendias, 2000). Recent molecular studies have further elucidated that Cd disrupts photosynthetic electron transport chains more severely than Pb by targeting photosystem II reaction centers (Gupta et al., 2024; Jin et al., 2025). Anatomical evidence suggests that approximately 56% of accumulated Cd binds to plant cell walls, restricting cellular expansion and metabolic functioning (Wierzbicka et al., 2007). In contrast, nearly all Pb (97.6%) is sequestered in insoluble forms within plant tissues, reducing its physiological mobility and toxicity (Wierzbicka et al., 2007). Pb accumulates within vesicles derived from endoplasmic reticulum fragmentation, suggesting rapid metabolic processing in epidermal cells that contributes to its comparatively reduced phytotoxicity (Wierzbicka et al., 2007).

The observed growth inhibition under metal stress is consistent with previous reports that Pb significantly suppresses root and shoot elongation, germination parameters, and dry biomass accumulation (Amoroso et al., 2001; Kiran et al., 2022), with Cd showing comparable or greater inhibitory effects (Ahmad et al., 2012). The reduction in root length under Cd stress is associated with decreased mitotic activity in root meristems (Xue et al., 2013; Wei et al., 2005) and irreversible inhibition of proton pumps essential for cell expansion (Aidid and Okamoto, 1993). Beyond these general metal toxicity patterns, the magnitude of growth inhibition varied substantially among species, revealing distinct tolerance capacities.

### 4.2 Species-specific responses and phytoremediation suitability

Clear species-specific differences emerged from this study, with *E. citriodora* demonstrating superior tolerance across all measured parameters. This species consistently maintained growth, accumulated higher biomass, and exhibited lower phytotoxicity even at the highest metal concentrations. The superior growth performance and tolerance observed in *E. citriodora* suggest that it possesses adaptive mechanisms enabling sustained growth under Cd and Pb stress. Although the present study did not quantify metal accumulation in plant tissues, these characteristics indicate that the species warrants further investigation as a candidate for phytoremediation. This metal-sequestration ability, combined with its robust biomass production under stress, positions *E. citriodora* as the most promising candidate among the four species for phytoremediation applications, consistent with previous reports on its potential for multi-metal contaminated sites (Patil et al., 2023). Recent field trials have confirmed *Eucalyptus* species as effective candidates for long-term phytoremediation of multi-metal contaminated sites, with *E. citriodora* showing particular promise due to its extensive root system and high biomass production (Akinbile and Mbohwa, 2025).

*E. camaldulensis* exhibited the highest shoot and root phytotoxicity values, indicating possible metal tolerance without succumbing to toxicity, a desirable trait for phytoremediation. Higher phytotoxicity indicates greater growth inhibition and physiological sensitivity under heavy metal stress. This species demonstrated strong potential for environmental remediation by accumulating substantial amounts of metals while maintaining sufficient growth (Gomes et al., 2012; Madejón et al., 2017; Motesharezadeh et al., 2017). The high phytotoxicity in *E. camaldulensis* roots under Cd treatment (81.82%) can be attributed to increased formation of oxygen-free radicals, accompanied by reduced enzymatic and non-enzymatic antioxidant activity (Asati et al., 2016), manifesting as root browning, leaf chlorosis, and discoloration (Kiran et al., 2022).

*E. tereticornis* and *E. globulus* occupied intermediate positions, with *E. tereticornis* showing relatively superior tolerance to Pb stress, possibly due to association with efficient sequestration mechanisms reported in previous studies. Their close clustering in hierarchical analysis suggests similar physiological strategies for coping with metal stress. **Supplementary Table 1–4** present the percentage change in the measured growth and physiological parameters of the four *Eucalyptus* species under different cadmium (Cd) and lead (Pb) treatments relative to their respective control plants. However, since Eucalyptus species inherently differ in growth characteristics, interpretation of absolute growth values should be considered together with phytotoxicity (%) and tolerance index (%), which normalize treatment responses relative to their respective controls.

The hierarchical clustering sequence, *E. citriodora* > *E. camaldulensis* > *E. tereticornis* > *E. globulus*, provides a clear ranking for species selection based on phytoremediation potential. The superior performance of *E. citriodora* may indicate greater tolerance to Cd and Pb stress. Previous studies have suggested that *Eucalyptus* species can immobilize or compartmentalize heavy metals, although these mechanisms were not investigated in the present study. This differential performance was further reflected in how each species allocated resources between shoots and roots under metal stress.

The superior tolerance of *E. citriodora* may be associated with several physiological mechanisms reported in previous studies, including enhanced antioxidant enzyme activity, efficient sequestration of heavy metals within vacuoles and cell walls, synthesis of phytochelatins and metallothioneins, maintenance of photosynthetic pigments, and improved nutrient homeostasis. Although these mechanisms were not experimentally examined in the present study, they provide plausible explanations for the observed differences among species and warrant detailed biochemical and molecular investigations in future work.

### 4.3 Shoot-to-root biomass partitioning strategies

Variations in shoot-to-root ratios reflect species-specific biomass partitioning strategies under stress conditions. *Eucalyptus tereticornis* exhibited preferential shoot investment under high Pb stress (shoot: root ratio of 1.46), whereas *E. camaldulensis* allocated more biomass to roots under high Cd stress (lowest ratio of 0.73), suggesting enhanced root development for metal tolerance. These patterns align with previous findings that the shoot-to-root ratio is modulated by species-specific strategies, developmental stage, and environmental factors, including metal stress (Ahmad et al., 2012). The observed declines in shoot-to-root ratios under elevated metal exposure are further supported by the established relationship between shoot/root length ratio and heavy metal accumulation in seedling tissues (Losi et al., 1994). Beyond these growth parameters, photosynthetic pigment content provided critical insights into the physiological basis of differential metal tolerance among the studied species.

### 4.4 Chlorophyll content as a stress indicator

Total chlorophyll content proved to be a reliable indicator of metal stress and species tolerance. The hormetic response observed in *E. tereticornis* and *E. citriodora* under low Pb (100 mg kg^-^¹) with chlorophyll increases of 11.96% and 2.13%, respectively, suggests that sub-toxic Pb concentrations may trigger compensatory antioxidant synthesis and stress response mechanisms (Dubey, 2011; El-Khatib et al., 2020). This biphasic response was unique to Pb and not observed with Cd, further confirming Cd’s higher toxicity even at low concentrations.

Cd-induced chlorophyll degradation occurs through replacement of central magnesium ions in chlorophyll porphyrin rings and inhibition of δ-aminolevulinic acid dehydratase, a critical enzyme in chlorophyll biosynthesis (Hernandez et al., 1996; Hopkins and Hüner, 2008; Villiers et al., 2011; Cenkci et al., 2010). Pb operates through similar mechanisms, including inhibition of essential element uptake and damage to photosystem protein complexes (Seregin and Ivanov, 2001). The higher chlorophyll retention in *E. citriodora* under both metal stresses is likely attributed to more efficient antioxidant enzyme systems (catalase and peroxidase) that scavenge reactive oxygen species and protect photosynthetic machinery (Xue et al., 2013). Advanced metabolomic analyses have recently identified specific antioxidant compounds, including flavonoids and phenolic acids, that confer enhanced Cd tolerance in metal-resistant *Eucalyptus* genotypes (Guarino et al., 2014; Zafar et al., 2022; Islam et al., 2025). Previous investigations have attributed chlorophyll preservation under heavy metal stress to enhanced antioxidant defense systems. Although antioxidant enzymes were not measured in the present study, these mechanisms may explain the observed responses. Recent work demonstrates that organic amendments can enhance stress tolerance in sensitive species like *E. camaldulensis* by improving nutrient uptake and strengthening antioxidant defenses (Ouyang et al., 2024). The interrelationships among these physiological responses were further elucidated through correlation analysis.

### 4.5 Correlations and implications for phytoremediation

The strong positive correlations between total chlorophyll and biomass parameters indicate that chlorophyll content is closely associated with plant growth under heavy metal stress due to improved photosynthetic efficiency and carbon assimilation (Taiz et al., 2015), supporting greater biomass accumulation. The negative correlation with phytotoxicity reinforces that metal-induced stress reduces chlorophyll synthesis and accelerates pigment degradation (Parmar et al., 2013). Emerging research emphasizes the importance of multi-trait assessment frameworks for selecting optimal phytoremediation candidates, with chlorophyll fluorescence parameters now recognized as rapid, non-destructive indicators of metal stress (Dadea et al., 2017; Bai et al., 2021).

The strong correlation between *E. tereticornis* and *E. globulus* (r = 0.994 under Cd; 0.988 under Pb) suggests similar physiological response mechanisms, despite their differing tolerance levels. This clustering pattern, combined with the species ranking, provides a practical framework for selecting appropriate *Eucalyptus* taxa for future phytoremediation studies, *E. citriodora* for multi-metal contaminated sites requiring both tolerance and biomass production, and *E. camaldulensis* for sites where maximum metal uptake is prioritized despite higher phytotoxicity symptoms.

### 4.6 Study limitations

The present study evaluated the comparative tolerance of four *Eucalyptus* species under controlled pot conditions using growth, biomass, chlorophyll content, phytotoxicity, and tolerance indices. Direct measurements of Cd and Pb accumulation in plant tissues, soil metal availability, antioxidant enzyme activities, and molecular responses were beyond the scope of this investigation. Consequently, conclusions regarding phytoextraction, phytostabilization, or the underlying physiological mechanisms should be interpreted cautiously. Furthermore, the experiment was conducted under controlled greenhouse conditions for 120 days using seedlings grown in sterilized red soil. Therefore, long-term field studies incorporating soil characterization, metal accumulation analyses, and multi-season evaluations are required before making definitive recommendations for large-scale phytoremediation programs.

### 4.7 Environmental implications

The findings of this study have direct and practical implications for the remediation of metal-contaminated soils in tropical and subtropical regions. The clear species ranking established, *E. citriodora* > *E. camaldulensis* > *E. tereticornis* > *E. globulus*, provides land managers and restoration practitioners with an evidence-based framework for promising candidate selection in phytoremediation programs that warrants evaluation under long-term field conditions.

For cadmium-contaminated sites: *Eucalyptus citriodora* may be considered for further field evaluation as it has emerged as the most suitable candidate due to its exceptional tolerance (maintaining 77.86% tolerance index at 100 mg kg^-^¹ Cd), sustained chlorophyll content (1.41 mg g^-^¹), and robust biomass production even under severe stress at initial phase. These traits make it ideal for phytoremediation of Cd from moderately to heavily contaminated agricultural soils affected by phosphate fertilizer overuse or industrial emissions.

For lead-contaminated sites: Both *E. citriodora* and *E. tereticornis* demonstrate strong potential. *E. citriodora* achieved 86.09% tolerance index at 250 mg kg^-^¹ Pb, while *E. tereticornis* exhibited effective Pb accumulation in roots (61.57% root phytotoxicity), suggesting suitability for phytostabilization applications where metal immobilization in root systems is desired.

For multi-metal contaminated sites: *Eucalyptus citriodora* may be one of the unequivocal choices, showing superior performance under both Cd and Pb stress, simultaneously a common scenario in industrial and agricultural landscapes affected by multiple contamination sources.

Beyond species selection, the strong correlation between chlorophyll content and biomass parameters (r = 0.994) validates chlorophyll fluorescence as a rapid, non-destructive monitoring tool for assessing plant health and remediation progress in the field. This enables cost-effective, real-time evaluation of phytoremediation effectiveness without destructive harvesting.

The deep root systems and high biomass production of eucalypts also contribute to additional ecosystem services, including carbon sequestration, soil erosion control, and microclimate improvement in degraded landscapes. For sensitive species like *E. camaldulensis*, which exhibited high metal uptake despite toxicity symptoms, soil amendments such as organic fertilizers could enhance tolerance and improve remediation outcomes, as demonstrated in recent studies (Ouyang et al., 2024). These findings collectively advance the development of sustainable, nature-based solutions for restoring metal-polluted environments, protecting adjacent agricultural lands from metal migration, and safeguarding human communities dependent on healthy soil ecosystems.

A limitation of the present study is that direct quantification of Cd and Pb accumulation in root and shoot tissues was not performed. Consequently, indices such as the Bioconcentration Factor (BCF), Translocation Factor (TF), and metal partitioning could not be determined. Therefore, the present work should be regarded as a comparative assessment of tolerance and early growth responses rather than a complete evaluation of phytoremediation efficiency. Future investigations integrating elemental analysis with physiological, biochemical, and molecular approaches will provide a more comprehensive understanding of species suitability for phytoremediation.

## 5 Conclusion

This comprehensive evaluation demonstrates that *Eucalyptus citriodora* demonstrated superior tolerance under controlled experimental conditions and therefore represents a promising candidate for future phytoremediation research. However, confirmation of its remediation efficiency requires field validation together with quantitative analyses of metal accumulation, translocation, and long-term plant performance. The species ranking established through hierarchical clustering (*E. citriodora* > *E. camaldulensis* > *E. tereticornis* > *E. globulus*) provides evidence-based guidance for species selection in phytoremediation programs. Cd consistently exhibited greater phytotoxicity than Pb across all parameters, reflecting its higher bioavailability and stronger interference with physiological processes. The strong correlations between chlorophyll content and growth parameters indicate its utility as a rapid, non-destructive indicator of metal stress. These findings provide a robust scientific foundation for selecting appropriate *Eucalyptus* species in phytoremediation programs, thereby advancing sustainable, nature-based solutions for restoring metal-contaminated soils and safeguarding ecosystem health in polluted landscapes.

## Data Availability

The original contributions presented in the study are included in the article/**Supplementary Material**. Further inquiries can be directed to the corresponding author.
