# Supplementary material for: Species-specific cadmium and lead tolerance in four Eucalyptus seedlings: implications for phytoremediation of metal-contaminated soils
Source: Front Plant Sci. 2026 Aug 21;17:1908520. doi: 10.3389/fpls.2026.1908520 (PMC13541658; doi:10.3389/fpls.2026.1908520)
Supplement: Supplementary file 1 [file DataSheet1.pdf]

Supplementary Material

1 Supplementary Data

Supplementary Tables S1–S4 present the percentage change in the measured growth and physiological parameters of the four *Eucalyptus* species (*Eucalyptus tereticornis*, *E. camaldulensis*, *E. globulus*, and *E. citriodora*) under different cadmium (Cd) and lead (Pb) treatments (T1 - Cd 25 mg kg^{-1}, T2 - Cd 50 mg kg^{-1}, T3 - Cd 100 mg kg^{-1}, T4 - Pb 100 mg kg^{-1}, T5 - Pb 125 mg kg^{-1} and T6 - Pb 250 mg kg^{-1}) relative to their respective control plants. The parameters include shoot length (SL), root length (RL), shoot-to-root ratio (S:R), collar diameter (CD), total dry biomass (TDB), shoot dry biomass (SDB), root dry biomass (RDB), shoot-to-root dry biomass ratio (S:R DB), and total chlorophyll content. Percentage changes were calculated to facilitate comparison of treatment-induced responses within each species across all metal concentrations without altering the original measured values. These supplementary tables provide the detailed dataset supporting the comparative analyses presented in the main manuscript.

1.1 Supplementary Tables

Supplementary Table 1. Percentage change analysis of different growth parameters in *Eucalyptus tereticornis* under different treatments.

Growth Parameters	T1	T2	T3	T4	T5	T6
SL	-25.91	-44.66	-50.05	-9.01	-16.53	-23.31
RL	-19.28	-26.51	-30.24	-5.42	-7.71	-31.81
S:R	-8.13	-24.59	-28.24	-3.77	-9.38	12.66
CD	-13.48	-25.17	-33.48	-2.92	-12.81	-18.43
TDB	-12.96	-21.34	-47.52	-3.61	-24.49	-30.23
SDB	-10.04	-14.33	-45.18	-0.73	-24.45	-30.12
RDB	-13.25	-35.02	-42.27	2.84	-11.67	-18.61
S:R DB	4.31	32.13	-4.89	-3.24	-14.21	-13.85
Chlorophyll	-10.11	-35.38	-44.03	12.17	-18.02	-27.18

Supplementary Table 2. Percentage change analysis of different growth parameters in *Eucalyptus camaldulensis* under different treatments.

Growth Parameters	T1	T2	T3	T4	T5	T6
SL	-40.08	-49.30	-67.43	-23.35	-28.16	-35.47
RL	-16.49	-35.19	-41.95	-4.42	-18.18	-18.83
S:R	-27.76	-21.22	-43.66	-19.44	-12.11	-20.46
CD	-26.65	-39.88	-57.72	-19.24	-16.43	-39.88

TDB	-28.40	-40.61	-49.68	-17.58	-28.04	-35.77
SDB	-24.95	-36.49	-46.91	-16.19	-27.84	-35.98
RDB	-98.12	-98.45	-98.63	-97.52	-97.81	-98.07
S:R DB	81.95	86.60	77.45	54.48	50.16	51.37
Chlorophyll	-31.32	-47.42	-64.58	-25.76	-40.85	-52.33

Supplementary Table 3. Percentage change analysis of different growth parameters in *Eucalyptus globulus* under different treatments.

Growth Parameters	T1	T2	T3	T4	T5	T6
SL	-34.38	-51.22	-60.76	-24.83	-32.38	-38.89
RL	-22.62	-27.98	-40.00	-14.29	-23.45	-30.48
S:R	-15.41	-32.55	-34.84	-12.53	-12.06	-12.34
CD	-32.93	-41.32	-65.27	-23.75	-40.12	-41.12
TDB	-24.27	-21.14	-42.61	-8.84	-14.31	-22.84
SDB	-27.42	-20.50	-44.78	-10.34	-15.05	-25.02
RDB	-17.11	-27.63	-37.50	-6.91	-14.80	-21.05
S:R DB	-12.47	9.85	-11.62	-3.67	-0.31	-5.08
Chlorophyll	-33.43	-37.41	-55.38	-21.74	-29.33	-47.13

Supplementary Table 4. Percentage change analysis of different growth parameters in *Eucalyptus citriodora* under different treatments.

Growth Parameters	T1	T2	T3	T4	T5	T6
SL	5.79	-9.13	-17.45	13.02	-0.36	-14.20
RL	5.95	-2.70	-20.00	14.92	5.19	-4.86
S:R	-0.04	-6.59	3.22	-1.67	-5.27	-9.74
CD	0.36	-6.24	-28.34	0.71	0.71	-11.41
TDB	0.63	-10.28	-22.14	0.73	-5.55	-13.91
SDB	0.78	-12.18	-23.87	0.42	-6.93	-9.58
RDB	0.88	-1.98	-15.42	1.54	-0.88	-29.52
S:R DB	-0.10	-10.40	-9.85	-1.10	-6.11	28.54
Chlorophyll	0.57	-11.43	-25.04	1.77	-14.51	-25.19
